# Survival analysis of bevacizumab plus taxane treatment in luminal metastatic breast cancer

**DOI:** 10.2144/fsoa-2020-0146

**Published:** 2021-01-19

**Authors:** Serafin Morales Murillo, Ariadna Gasol Cudos, Joel Veas Rodriguez, Carles Canosa Morales, Jordi Melé Olivé, Felip Vilardell Villellas, Douglas Rene Sanchez Guzman, Edelmiro Iglesias Martínez, Antonieta Salud Salvia

**Affiliations:** 1 Department of Oncology, Hospital Arnau de Vilanova de Lleida, Avenida Rovira Roure, 80, Lleida 25198, Spain; 2 Breast Cancer Unit, Hospital Arnau de Vilanova de Lleida, Avenida Rovira Roure, 80, Lleida 25198, Spain; 3 Pathology Unit, Hospital Arnau de Vilanova de Lleida, Avenida Rovira Roure, 80, Lleida 25198, Spain

**Keywords:** • bevacizumab, bevacizumab plus paclitaxel, chemotherapy, luminal, luminal breast cancer, metastatic breast cancer, overall survival

## Abstract

**Background:**

The treatment of luminal metastatic breast cancer is based on endocrine therapy and chemotherapy treatment is limited to the progression of this treatment.

**Materials & methods:**

We analyzed the efficacy of treatment with bevacizumab plus paclitaxel in 43 patients with hormone receptor-positive and HER2-negative metastatic breast cancer.

**Discussion:**

Paclitaxel plus bevacizumab combination is a useful treatment in metastatic luminal breast cancer with an impressive overall survival of 31 months, similar to combination to endocrine therapy and targeted therapy in first line. In patients with hormone resistance, endocrine therapy saw worse results thus the taxol plus bevacizumab combination could be a better option. This combination does not influence the results of subsequent treatments; therefore, it could provide a good option for patients.

## Lay abstract

Luminal metastatic breast cancer often needs endocrine therapy. However, there is a subgroup with worse prognosis where endocrine therapy lacks benefit. This subgroup needs an optimal chemotherapy regimen. In our series we show a benefit in this subgroup with the bevacizumab plus taxol combination, with a good tolerability profile and improved overall survival.

Luminal breast cancer (hormone receptor-positive and HER2-negative) is the most common subtype of metastatic breast cancer (MBC) [1], with a favorable prognosis compared with other subtypes of MBC [1]. The estrogen receptor signaling pathway is the main driver of cancer cell growth and survival in these tumors, so endocrine-based therapies are considered the most effective treatments [1]. There were several randomized controlled trials to find innovative therapies with new targeted drugs combined with hormone treatment to improve the results with endocrine treatment. The most relevant examples of these new targeted therapies are the mTOR inhibitor everolimus and the cyclin-dependent kinase (CDK) 4/6 inhibitors palbociclib, ribociclib and abemaciclib, which are used in combination with endocrine therapies achieving significant improvements in disease-free survival (DFS), specifically in the first line. These improvements have led them be the reference in the first line in this breast cancer subtype [2–5].

The so-called IMELDA scheme [6], with the sequence of treatment with chemotherapy with taxanes plus bevacizumab followed by a maintenance scheme with the combination of capecitabine with bevacizumab, leads to an impressive overall survival (OS) that reaches 3 years. However, comparative studies between this treatment regimen and the combination of endocrine treatment are still lacking. As a result decisions as to the best option can be controversial.

Over the last few years angiogenesis has become implicated in cancer pathophysiology and studies into its mechanisms have increased. This has led to the first anti-angiogenesis drug, bevacizumab, becoming commonly used in the treatment of colorectal, renal cell and brain cancers. Bevacizumab is a humanized monoclonal antibody targeting the VEGF-A. It is delivered in combination with chemotherapy and has consistently shown clinical efficacy in the treatment of MBC [7].

Bevacizumab was approved following the ECOG 2100 trial, which demonstrated that it, in combination with paclitaxel, as a first-line treatment for advanced breast cancer nearly doubled the time to progression and tumor response rate [8]. Other Phase III trials have also revealed a smaller absolute improvement in DFS and response rates, although none have demonstrated enhanced survival as yet. This has led to controversy and debate over the use of bevacizumab [9]. In addition, a meta-analysis of three randomized, controlled, Phase III trials confirmed that the addition of Bevacizumab to chemotherapy regimens provides substantial benefit for women with MBC in terms of DFS and objective response, although not in OS [10].

We reviewed a retrospective series of 43 patients in a single institution with hormone receptor-positive and HER2-negative MBC treated with bevacizumab plus paclitaxel with the aim of describing the clinical efficacy with regards to survival.

## Materials & methods

### Clinical data

We analyzed the survival efficacy of a retrospective series of 43 patients with luminal MBC treated with bevacizumab plus paclitaxel in a single institution as a conventional treatment between September 2010 and September 2019.

All patients had a histologically proven diagnosis of breast cancer. HER2/neu receptor status was evaluated using immunohistochemistry or fluorescence *in situ* hybridization. HER2/neu oncoprotein expression negativity was assessed using the Hercep test, scoring 0 (absent) or 1+ (weak), and the negativity of the HER2/neu gene amplification was confirmed by fluorescence in situ hybridization if score was 2+ (moderate) in the Hercep test. The expression of hormonal receptors was assessed by histoscore and it was considered as positive if the estrogen receptor expression was superior to 0. Finally, patients selected was HER2/neu receptor-negative and estrogen receptor-positive.

### Follow-up

Patients were followed until September 2019. Assessment of survival was calculated from the starting date of treatment with bevacizumab until progression, the date of last visit or death. Treatment with paclitaxel plus bevacizumab was performed until progression or unacceptable toxicity, and then some patients followed a maintenance schema with capecitabine plus bevacizumab or endocrine therapy only.

### Statistical analysis

Descriptive statistics with 95% CI were calculated according to standard procedure. Survival curves were constructed using the Kaplan–Meier method. The log-rank test was used to compare survival curves. The test was conducted at a 5% significance level.

## Results

The median age of patients was 48 years (range: 31–80 years), 15 patients (35%) were metastatic at initial diagnoses, 17 (40%) progressed during the first 5 years of adjuvant endocrine therapy and the remaining 11(25%) progressed after finishing adjuvant endocrine therapy. The majority of the patients received adjuvant treatment (24 out of 28) and a total of 12 patients received previous endocrine therapy for first-line metastatic disease. The most frequent metastasis site was: bone (34), liver (17) and lung (15), but only bone metastasis was present in four patients. The clinicopathological characteristics are indicated in Table 1.

**Table 1.** Clinicopathological characteristics of patients.

Median age	48 years
Disease-free interval	
0	15 (35%)
1–60	17 (39%)
>60	11 (26%)
Previous adjuvant treatment (28 patients)	
Chemotherapy and hormonotherapy	24 (86%)
Hormonotherapy	3 (10%)
None	1 (4%)
Previous treatment in metastatic line	
Hormonotherapy	12 (28%)
None	31 (72%)
Metastasis site	
Bone	34
Liver	17
Lung	15
Only bone	4

After a total of 259 cycles administered with an average number of six (range: 2–16), we found a DFS of 13 months (95% CI: 9.5–16.4) and OS of 31 months (95% CI: 23.4–38.5) counted from the first treatment performed with taxol plus bevacizumab. In patients whose initial diagnosis was metastatic disease the DFS and OS was 20 (range: 13–28 months) and 57 months (range: 36–72 months) while for the patients who progressed during adjuvant treatment the DFS and OS were 12 months (range: 9–15 months) and 19 months (range: 13–28 months), respectively, and for patients with >60 months of appearance of metastasis the DFS and OS was 21 months (range:12–30 months) and 36 months (range: 27–45 months), respectively.

We also analyzed the survival efficacy of taxol plus bevacizumab according to previous treatment with hormonotherapy in the first line of metastatic disease. We found a similar OS counting from the first instance of metastatic disease with 40 months (range: 25–54 months) versus 32 months (range: 23–40 months) for patients with previous treatment with hormonotherapy in first line of metastatic disease compared with no previous treatment (log rank p: 0.161). DFS was also similar in both groups with 11 versus 14 months for previous or no previous treatment, respectively (log rank p: 0.36).

We could consider that patients that progressed to taxol plus bevacizumab could have a poor prognosis and might have a bad response to subsequent treatments. In our series the survival postprogression to taxol plus bevacizumab was similar between the group of patients that received a previous first line with endocrine therapy than patients that the initial treatment was taxol plus bevacizumab (log range p: 0.705).

**Figure 1.**
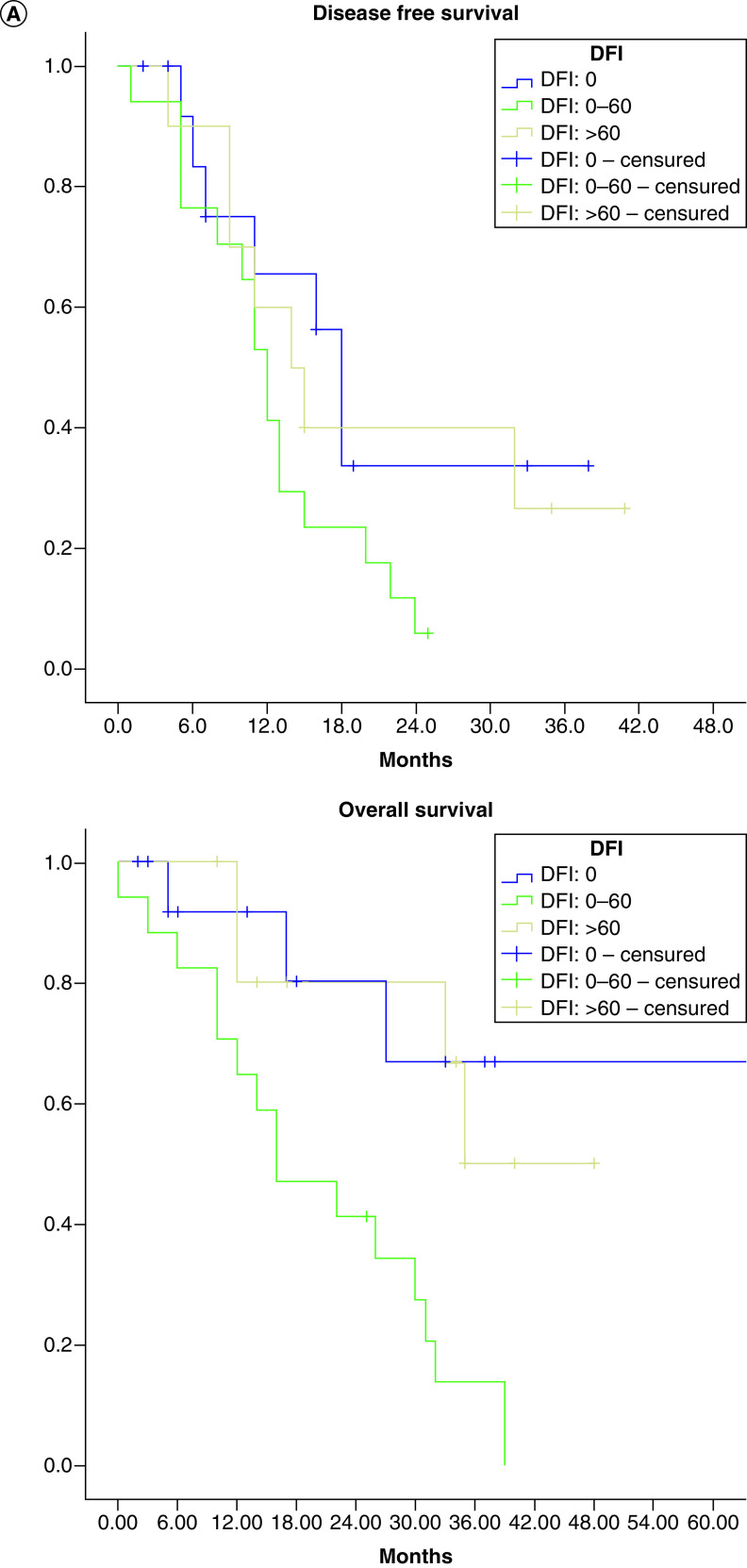

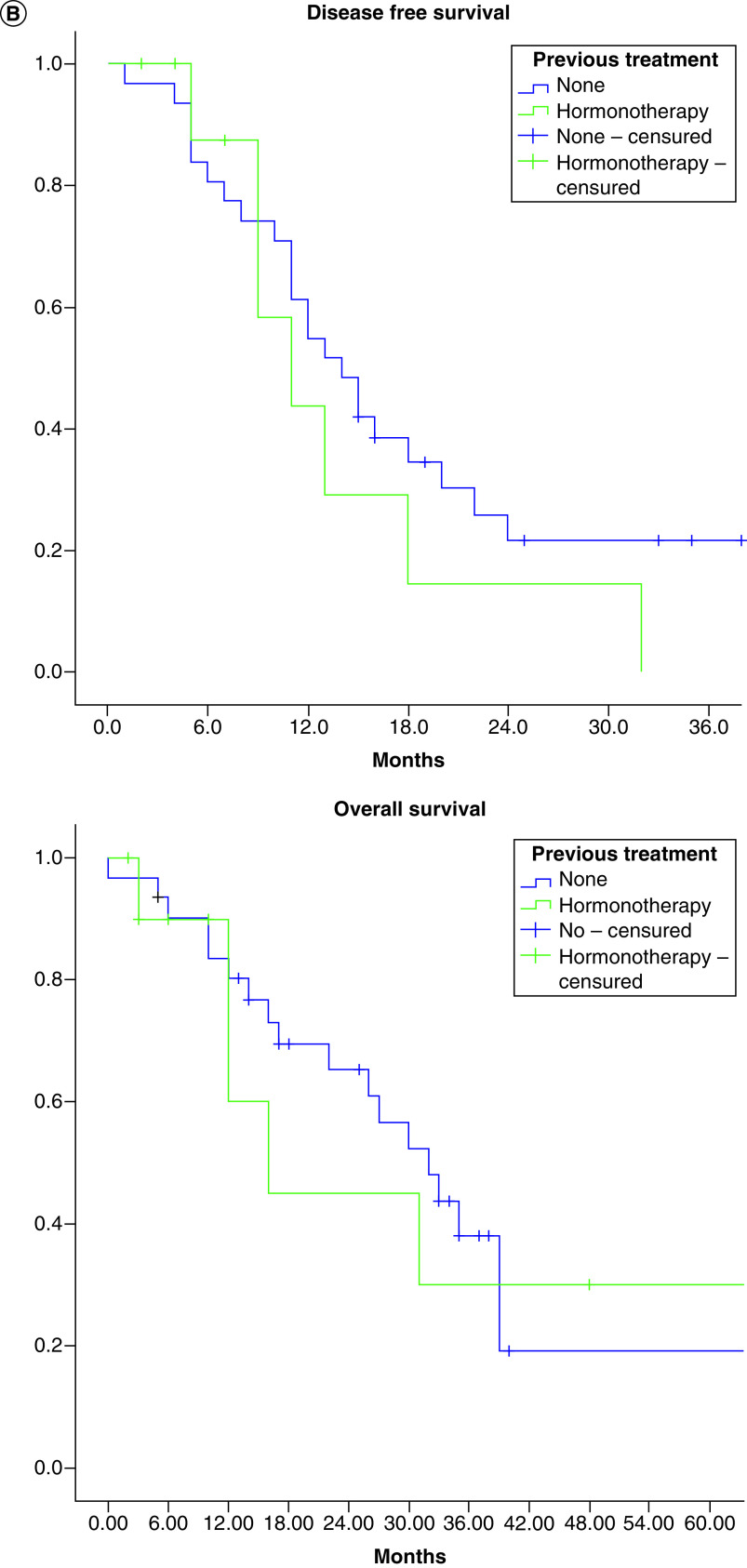

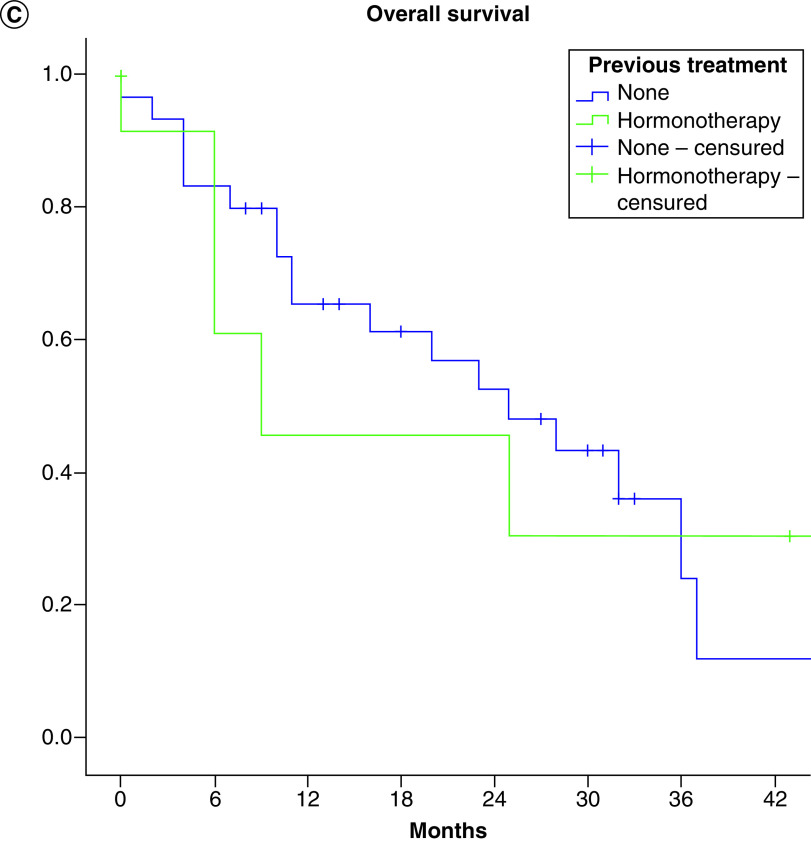
Survival curves. **(A)** According interval of metastatic disease. **(B)** Survival curves according previous treatment with hormonotherapy in the first line of metastatic disease. **(C)** Survival after treatment with taxol plus bevacizumab in patients with previous hormonotherapy or not. DFI: Disease free interval.

In Figure 1A we represent survival curves according to the interval of occurrence of metastatic disease, in Figure 1B we represent survival curves according to previous treatment with hormonotherapy in the first line of metastatic disease, and in Figure 1C we represent survival after treatment with taxol plus bevacizumab in patients with and without previous endocrine therapy in the first line metastatic disease.

## Discussion

We achieved a median OS of 33 months in the global series in the range of the majority of trials. In the IMELDA study [6] the median OS was 39 months but in the group selected to take maintenance treatment, in the hormone receptors (HR)-positive patients, the median DFS was 13 months, similar to our series. Recently the B-Share trial [11] reported a median OS of 28.2 months in the HR-positive group treated in first line from a total of 345 patients with 40% treated previously with endocrine therapy in the first line. These results contrast with only 28% of patients previously treated with endocrine therapy in our series. In the GINECO group study [12] where the primary objective was to assess the efficacy of exemestane plus bevacizumab as a maintenance therapy after first-line taxane and bevacizumab combination, we found a similar OS with 26–32 months in a HR-positive population with 30% of patients treated previously with endocrine therapy. In another observational study, Dieras *et al.* found similar results in OS with a median of 41 months with a proportion of patients with only a 14% of previous treatment with hormonotherapy [13]. Finally, in the multinational ATHENA study [14] with 1430 HR-positive patients the median OS ranges from 17.4 to 38.8 months according to different prognostic factors.

Recently the combination of hormonotherapy with CDK 4/6 inhibitors has shown a good survival efficacy, especially in the first line of metastatic disease, and has been established as the best treatment in international guidelines [15]. The lack of direct comparative studies between chemotherapy and endocrine therapy plus CDK 4/6 inhibitors suggests that indirect studies have to be carried out, which usually involve many errors given the heterogeneity of these studies [16]. Giuliano *et al.* published an interesting meta-analysis reviewing the efficacy between endocrine therapy and chemotherapy [17]. Their results suggest that endocrine therapies plus targeted therapies remain the best treatment choice, since chemotherapy was not shown to be better than endocrine therapy with targeted agents even when using highly active chemotherapy regimens. However, when comparing with paclitaxel plus bevacizumab, a higher proportion of patients achieving an overall response have been observed with this chemotherapy combination.

Since the cohort of estrogen receptors (RE)-positive patients is too heterogeneous to do comparative studies of different treatments, we need to analyze the clinical benefit in the different subgroups. One of the most interesting subgroups would be patients with debut metastasis, because we do not know their natural history and their sensitivity to different treatments. In our series, the 10 patients with debut metastatic initially treated with taxol plus bevacizumab had a median DFS of 38 months (95% CI: 21–54) which is consistent with studies treating with endocrine plus targeted therapy such as PALOMA 3 [18], with a median DFS of 27.9 months in the subgroup of novo metastatic; or MONARCH 3 [19] with 28.18 months – in this study, 40% of patients were novo metastatic. Recently, the results of the PARSIFAL study were presented at ASCO 2020, finding specifically in the *de novo* metastasis group a median DFS of 31.6 months [20].

In this cohort of patients, the tolerability and applicability of this regimen is clinically feasible. We did not find any grade 3 adverse events, and the median number of cycles was six, which means a good tolerance; and patients who received fewer cycles were because of rapid progression, which made these patients unable to receive later treatments.

Another more frequent scenario is patients’ progressing after first line with endocrine therapy. Actual guidelines recommend initial treatment with endocrine therapy in the majority of the patients [15], and after progressing there are two options: to continue with endocrine therapy if there is a good response to the first line (normally with duration of response >6 months or starting with chemotherapy treatment as second option [21]. In our series, 12 patients were initially treated with endocrine therapy and their DFS from the start of the chemotherapy treatment was 16 months (95% CI: 10.7–21.2). In contrast, Aapro *et al.* in the NorBreast-231 trial, which investigated the survival with chemotherapy (taxol or vinorelbine oral) in this context, found a DFS similar in both arms of only 6.4 months [22]. The results with second line endocrine therapy are also disappointing, with an approximate DFS of 7 months with fulvestrant [23,24], and with the combination of fulvestrant with targeted therapy with a significant DFS around 11–16 months [25,26]. Recently, the pearl study demonstrated similar results in DFS (9–11 months) among the combination with endocrine therapy and palbociclib and treatment with chemotherapy with capecitabine in second-line treatment [27].

Patients with a short metastatic disease-free interval had the worst prognosis in the Athena study [15]. Most of these patients are on adjuvant hormonal treatment and have a relapse during it, so having a primary endocrine resistance the response to a second endocrine therapy line will be disappointing. In the Monaleesa trial, the DFS was 13 months in the combination of ribociclib plus fulvestrant versus 8 months in the fulvestrant arm [28]. In contrast, for the 14 patients in this context found in our series, the DFS was 16 months.

We must also consider that the treatment of metastatic cancer of the luminal phenotype is based on the sequencing of several treatments so it is important to know the effect of survival after a line of treatment. The standard use of the combination of endocrine therapy plus CDK 4/6 inhibitors has encouraged the analysis of survival after progression to this treatments and the effect of subsequent treatments [29]. The timing of the survival after progression to endocrine therapy plus CDK 4/6 inhibitors were similar and independent to the first treatments in the Turner analysis [30]. We analyzed the survival after progression on taxol plus bevacizumab and found a median OS of 25 months independent of previous treatment with endocrine therapy, and thus treatment combination does not appear to influence the results of the following treatments.

## Conclusion

The paclitaxel plus bevacizumab combination is a useful treatment in metastatic luminal breast cancer with an impressive OS of 33 months. Although the clinical guidelines consider endocrine treatment as the best option in the first line, the effectiveness of this treatment has not been overcome yet by the combination of paclitaxel plus bevacizumab.

In patients with endocrine resistance the subsequent endocrine therapy give the worst results with median DFS around 6–10 months. In this scenario, the taxol plus bevacizumab combination could improve these results; in our series we achieved 16 months. Although our series is not long enough to be robust with conclusions, the lack of comparative studies gives us a sing of better effectivity.

Finally, the selection of this treatment combination does not influence the results of subsequent treatments regardless of which line is indicated, so it should be a good option to take into account in the treatment of MBC with luminal phenotype.

## Future perspective

Chemotherapy treatment in metastatic luminal breast cancer patients is limited to progression after endocrine therapy or patients with primary endocrine resistance. We need to analyze the results of this treatment in different contexts than endocrine treatment in the metastatic line. In the next few years the principal treatment for metastatic luminal breast cancer will likely be ET and chemotherapy will be limited to patients with endocrine resistance and poor results with endocrine therapy (ET). In our series we achieved an impressive DFS of 16 months, and in patients with progression after ET a DFS of 11 months. New agents would be compared with these results to improve the OS in these poor prognosis patients.

**Summary points**

- We describe the efficacy of paclitaxel plus bevacizumab in a cohort of patients endocrine resistant because of a progression to previous hormonotherapy or clinical criteria to first-line chemotherapy (early relapse and metastatic visceral disease).
- Patients were selected with an adequate performance status to be able to receive at least two cycles of this combination. We achieved a disease-free survival of 16 months and an impressive overall survival of 33 months.
- We believe that metastatic luminal patients with endocrine resistance should be considered for chemotherapy with the most effective combination given its poor prognosis and, currently, taxol plus bevacizumab has displayed the best results.
